# Estimating the Health Risk Associated with the Use of Ecological Sanitation Toilets in Malawi

**DOI:** 10.1155/2017/3931802

**Published:** 2017-11-08

**Authors:** Save Kumwenda, Chisomo Msefula, Wilfred Kadewa, Bagrey Ngwira, Tracy Morse

**Affiliations:** ^1^College of Medicine, University of Malawi, P/Bag 360, Chichiri, Blantyre 3, Malawi; ^2^The Polytechnic, University of Malawi, P/Bag 303, Chichiri, Blantyre 3, Malawi; ^3^Sanitation and Hygiene Applied Research for Equity (SHARE), London School of Hygiene and Tropical Medicine, London, UK; ^4^Lilongwe University of Agriculture and Natural Resources, P.O. Box 219, Lilongwe, Malawi; ^5^Department of Civil and Environmental Engineering, University of Strathclyde, Glasgow, UK

## Abstract

Use of Ecological Sanitation (EcoSan) sludge is becoming popular due to increasing price of organic fertilizers in Malawi; however, there is little evidence on the associated risks. Quantitative microbiological risk assessment (QMRA) was done to determine health risks associated with use of EcoSan. Pathogens considered included* Escherichia coli (E. coli)*,* Salmonella,* and soil transmitted helminths (STHs). Exponential and Beta Poisson models were used to estimate the risk from helminthic and bacterial pathogens, respectively. Main exposure pathways were through poor storage of sludge, contamination of foods during drying, walking barefoot on the ground contaminated with sludge, pit emptying without protection, and application of sludge in the fields. Estimated annual risk for* Ascaris lumbricoides, Taenia, and* hookworms was approximately over 5.6 × 10^−1^ for both Fossa Alternas (FAs) and Urine Diverting Dry Toilet (UDDTs). Risk from* E. coli* and* Salmonella* was 8.9 × 10^−2^ and above. The risks were higher than WHO acceptable risk for use of faecal sludge in crops of 10^−4^ infections per year. Promoters and users of EcoSan latrines need to consider advocating for strict guidelines to reduce the risk.

## 1. Introduction

It is estimated that 2.7 billion people use on-site sanitation word-wide and of these 1.77 billion use some kind of a pit latrine. Of those using some form of pit latrines, 65% are found in Sub-Saharan Africa [1]. In the developed world, water closet toilet is the most common and came into use in the 1880s with the aim of reducing the transmission of sanitation related diseases especially cholera epidemics [2]. Sanitation facilities, especially improved ones, aim at reducing diseases transmission but both on-site and off-site sanitation have their challenges. Water closet toilets are challenged by problems in water supply, in design and operation, and in treating the sewage and pollution of the environment [3, 4]. As for traditional pit latrines, they have been reported to pollute ground water, easily collapse due to effect of underground water and sandy soils, and need more land as they do not operate on permanent basis and do not promote reuse of its content for agriculture [5]. Currently, there has been an emphasis on the use of Ecological Sanitation (EcoSan) as it appears to overcome most of the problems faced by the water closet toilets and the traditional pit latrines. EcoSan latrines have several benefits including the fact that the contents can be used to fertilize crops and that, compared to traditional pit latrines, environmental pollution (from leaching of the contents) is reduced [6, 7]. With the increasing cost of inorganic fertilizer, water shortages, and lack of available space for building pit latrines in periurban areas, the use of EcoSan latrines is a better option [8]. The use of EcoSan latrines is becoming popular in Malawi as households want to benefit from the faecal sludge produced by the latrines [9]. However, studies have shown that sludge produced from these latrines is unsafe for use in agriculture and increases the health risks to the communities [2, 10, 11].

**Figure 1:**
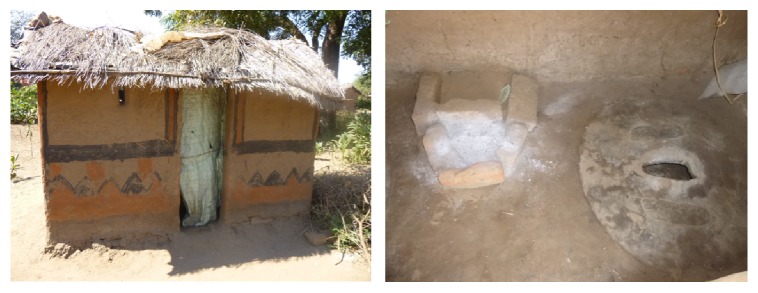
Fossa antenna superstructure and inside the latrine in Chikwawa.

**Figure 2:**
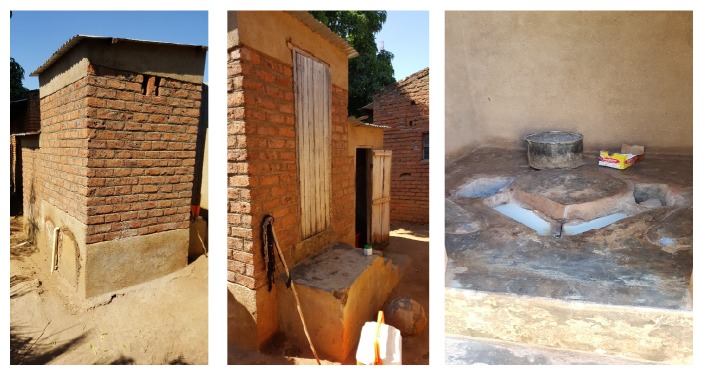
UDDT back view, front view, and inside showing urine diverting system and two drop holes, Blantyre, Malawi.

A “health risk” is defined as a factor that raises the probability of an adverse outcome relating to the wellbeing of a person or population [12]. In relation to the use of EcoSan, the application of pit contents (referred herein as EcoSan sludge) to agricultural fields has been reported to increase the risk of bacterial, viral, and helminthic infection through contact, accidental ingestion, and consumption of uncooked vegetables [13, 14]. Therefore, if not well managed, the use of EcoSan latrines and their pit contents may increase the transmission of diseases like diarrhoea and helminthiasis in the community. In Malawi, the most common types of EcoSan latrines available are Urine Diverting Dry Toilets (UDDTs) and Fossa Alternas (FAs). The UDDTs are more common in periurban areas and primary schools where people can access and afford to buy the necessary materials while, in rural areas, the most common EcoSan latrine type is the FA which can be constructed using local materials [10]. Figures 1 and 2 show the FA and UDDT, respectively.

The Ministry of Agriculture, Irrigation and Water Development in Malawi states that the FA is a simple twin pit system where usage alternates between the pits. The pits are permanent and use one movable slab. When newly built, one pit is covered by logs of trees or bamboo and the side with a slab is put to normal use. After each use, one adds a handful of ash and three hands full of dry soil into the pit [16]. When the pit in use is full (approximately after 6 months of use), it is covered with soil and then sealed. The slab is then moved to the second pit. The filled pit is left for six months so that the excreta can decompose after which the pit contents are removed for use [17]. The two pits are used interchangeably thereby making the EcoSan latrine permanent as it can be used over and over again. The FA has a permanent super structure and is normally made from bricks with mud mortar (Figure 1) and grass thatched roof [16]. The advantages of FA are that the use of ash and soil reduces smell and flies, it is cheap, and it can be used where land is scarce. The disadvantage are that the pits must be emptied regularly, and that the use of ash and soil is required, which is not always pleasant or easy for the user. The FA is the most common EcoSan latrine in Malawi [10]. The UDDT is a type of latrine built above the ground with double vaults for the collection of human excreta with the same operational principles as the FA, except that UDDTs have a urine diversion system [16].

Apart from bye-laws on use of sewage in the cities in Malawi, there are no regulations governing the use of EcoSan sludge. Harvested sludge from pits of EcoSan latrines is not always used for agriculture because owners of the latrines either do not have gardens or they feel disgusted by the sludge. For example, in periurban areas, the EcoSan sludge was often thrown away in places designated for general solid waste and sometimes in the rubbish pit around the household while, in the rural areas, sludge was disposed around the household surrounding. Previous efforts were made to promote the selling of EcoSan sludge to fertilizer companies and large scale farmers who grow nonfood crops but the sludge production from latrines could not meet the demand; hence the strategy was unsuccessful [18]. Moreover, the positive response shown by companies and large scale farmers demonstrated that if the campaign for EcoSan latrines can be intensified, they may be able to get the required supplies. The current situation where unused sludge is disposed of without following any regulations may increase the public health risks to the communities and calls for proper investigations.

Currently, several developing countries are trying to find suitable means of managing EcoSan sludge safely [19] with the primary concern being the presence of helminths especially the more resistant* Ascaris lumbricoides*. Conditions under which EcoSan operate may not meet conditions required for rapid* Ascaris* inactivation including a temperature of more than 40°C, less than 5% moisture content, and a pH of more than 12 [20]. A literature review done in 2014 showed that conditions for pathogen die-off vary in different countries and indicated the need for country specific information to be able to produce safe sludge [8]. However, such local data is often inadequate and this poses a challenge to producing safe sludge from EcoSan latrines [10, 19, 21]. Several studies have estimated the health risk of using urine, faecal sludge, and specifically sludge from EcoSan latrines and have found varying results due to differences in the epidemiological data, pathogen doses in the medium considered, and assumptions made. For example, studies done in Scandinavia found that the annual risk from* Ascaris* in faecal sludge after 12 months of storage ranged from lower than 10^−3^ to 2 × 10^−3^ [14, 22]. The risk is expected to be low because of the low concentration of helminths and weather conditions. In Mexico, the overall risk for consuming vegetables after fertilizing them with biosolids was found to range from 2 × 10^−1^ to 9 × 10^−5^ [23]. These studies show variations in risks which depend on local conditions. As such it is evident that specific estimations of risk are needed for Malawi because of the differences in environmental conditions and associated behaviours [24, 25].

“Health risk” in this study is defined as the likelihood of pathogens surviving in EcoSan sludge causing ill-health to people exposed. EcoSan, like any other sanitation technology, may, if not properly managed, pose a health risk to users and the general public. A previous study found that people may be exposed to pathogens through accidental ingestion during emptying the pit, application in the garden, contamination of water resources and crops, and children playing with soil [26].

Use of contaminated EcoSan sludge has been shown to pose a health risk in several studies [18, 19, 27]. For example, studies in South Africa and Nigeria isolated helminths ova from leaves of vegetables and pathogens from fruits, respectively, after they were fertilized with faecal sludge [19, 27]. As such, there is a proven need for clear and safe handling practices to be implemented to reduce transmission of diseases. Fossa Alternas (FAs) and Urine Diverting Dry Toilets (UDDTs) have been extensively promoted as Ecological Sanitation (EcoSan) latrine options in Malawi but little was known about whether they were used properly until a qualitative study of EcoSan users was conducted in periurban (Blantyre) and rural (Chikwawa) areas of Malawi in 2016. The study showed that blockages of urine diversion systems, intensive management, and maintenance needed for the latrines were major problems which affected attitudes about EcoSan use. Furthermore, use of soil and ash, urine diverting, use of hot water and chemicals to kill maggots, urinating in the drop-hole of the UDDTs, and poor maintenance of roof were some of the practices reported on use of these latrines [18]. These practices may lead to production of unsafe sludge from EcoSan toilets. Evidence that pathogens are not completely killed in EcoSan sludge has been reported in several studies [28–33]. In Malawi, sludge samples from EcoSan latrines in five districts were found to have viable helminthic ova and* E. coli* above the World Health Organization (WHO) standards for reuse of faecal sludge for agriculture [33, 34]. Though the assessment was not done to quantify the health risks that users of sludge and general public are exposed to, the study gave evidence of the risk especially with high prevalence of helminthic diseases in the country [35, 36]. Evidence from rural El Salvador showed that using EcoSan sludge which is not solar treated was associated with increased prevalence of enteric parasitic infections [32]. For the sludge to be safe, appropriate procedures during use should be followed, that is, adding at least one cupful of ash and three cups of soil after use including availability of conducive environmental conditions [6, 26, 37]. Despite some latrines meeting these conditions, it has been found that they may still produce unsafe sludge [38, 39]. Despite following all procedures during use of EcoSan latrines, it is important to consider EcoSan sludge as containing pathogens during emptying the pits and storage and during application in the field. The increasing use of EcoSan latrines and their sludge in Malawi therefore calls for thorough assessment to ensure the technology is safe and promotes public health. This study aimed to assess the health risk for using EcoSan sludge using Quantitative Microbiological Risk Assessment (QMRA) which involved the identification and quantification of the likelihood of hazards occurring resulting from use of EcoSan sludge taking into account the possible harmful effects on people using the sludge [40]. The resultant risk estimates will assist in the development of appropriate interventions to reduce the risks to recommended levels.

## 2. Method

### 2.1. Quantitative Microbiological Risk Assessment

Quantitative microbiological risk assessment (QMRA) was done to determine the health risks associated with use of EcoSan sludge in Blantyre (periurban) and Chikwawa (rural) in Malawi. In this study, the pathogens considered included pathogenic* E. coli*,* Salmonella,* and helminths (*Ascaris lumbricoides*,* Trichuris trichiura,* and hookworms). It is argued that when the adverse effects are due to microbial risk, then a QMRA is preferred to qualitative risk assessment and an epidemiological study because the two do not take into account the possibility of different doses that individuals may be exposed to [41].

### 2.2. The QMRA Process and Data Collection

**Figure 3:**
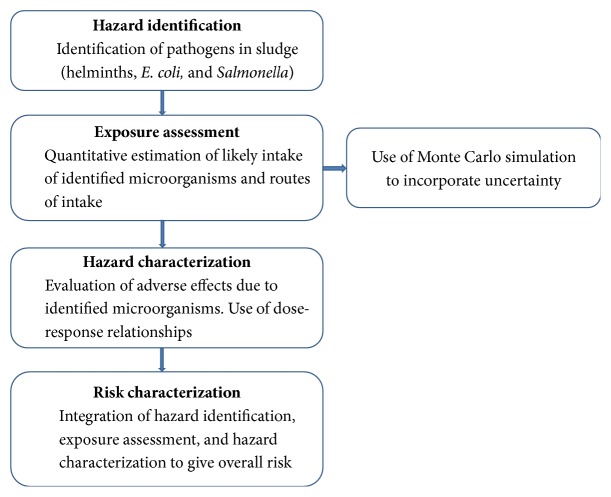
Steps followed during the QMRA adapted from Boone et al., 2010 [15].

The steps followed during the QMRA process were summarized in Figure 3.

Each step of the Figure 3 used data from laboratory analysis, observation, and literature review. Risk assessment followed the steps described below.

*Hazard Identification.* The hazards were identified from thirty-five (35) EcoSan latrines (13 FAs and 22 UDDTs) which were sampled after 12 months waiting period after the pits were sealed to determine the presence of hazards in the EcoSan sludge. The samples were collected from sludge immediately after being removed from the pit by taking from the top, middle, and bottom of the pile and then mixed in one container. Sample containers were kept in cooler box for transportation to the laboratory within 3 hours of collection, where they were kept in a refrigerator and processed the same day for bacteria, and within 2 days for helminths. Recovery of helminths from the latrine faecal matter was done using the modified triple floatation protocol [42]. An Olympus BX41 microscope was used to identify and enumerate the potential viable eggs. For* E. coli* and* Salmonella* 1 g of sludge sample was placed in a test tube and processed using standard operating procedures [43–45]. The hazards were defined as the pathogens recovered in the sludge after 12 months of storage and included bacterial pathogens and helminths. These were quantified and considered in the QMRA. The study also considered* Ascaris lumbricoides* because it is more resistant in sludge than all other helminths and is used as an indicator organism [23]. The bacterial pathogens were considered because of the high occurrence of diarrhoeal diseases in the population under consideration of which* Salmonella* and* E. coli* are the main reported causes [39, 46–48].

*Exposure Assessment.* Identification and analysis of the possible exposure pathways were done by direct observation. Checklist observation of pit emptying (*n* = 3) and sludge management (*n* = 35) was undertaken. The possibility of intake of STHs,* Salmonella,* and* E. coli* through sludge and the likely frequency of exposure was quantitatively assessed. The observations were done during use, emptying, storage, transportation, and application of sludge in the field. Results of the practices during use have been published elsewhere [18]. During observations, special attention was paid to possible ways where sludge can end up in the mouth of household members. Apart from oral route, other indirect means, for example, contamination of foods and water, were also assessed. The population likely to be exposed to the EcoSan sludge was noted. We used the estimated maximum doses used in other studies of 0.2 g of soil per day for children and 50% of the children's dose (0.1 g) of soil per day for adults to come up with the exposure doses [14]. The dermal contact with sludge was estimated to be three times that of ingestion and was estimated at 0.3 g depending on the practices observed, emptying with bare feet and bare hands and application in the fields. The reference population exposed was men, who were observed to be involved in almost all the activities involving EcoSan latrines in Malawi. These activities included construction, covering the filled pit, removal of sludge, transportation to the field, and application in the fields. Women and children were also involved but their observed exposure was lower than men. The maximum exposure periods took into consideration the common maximum survival times found in previous studies which reported 2 months for bacteria pathogens and 2 years for helminths [49]. The maximum survival times of concern were for helminths because they are usually longer than those for bacterial and viral pathogens. Since, in real life situation, not all individuals will be exposed and the exposure dose varies, Monte Carlo simulation was used to minimise uncertainties in the estimations of exposure doses. “Monte Carlo simulation is a statistical technique by which a quantity is calculated repeatedly, using randomly selected ‘what-if' scenarios for each calculation” [50]. Since exposures do not always occur in a consistent manner and individuals are exposed to different doses at different times, it was important to simulate the exposures and in total 10,000 simulations for exposure doses were made at a probability of between zero and one [51].

*Hazard Characterization*. Characterization was achieved by quantitative evaluation of adverse effects following exposure to hazards. The diseases that these pathogens cause were known and their prevalence was estimated from literature. Literature also provided information about the symptoms, severity, and death rates from the pathogens of focus and guided the study in making sure that all people who were likely to be at risk were identified. PubMed and Google Scholar were searched using keywords including prevalence, infection rate, symptoms and helminths,* Ascaris lumbricoides, Taenia,* hookworm,* E. coli*,* Salmonella*, population exposed, exposure doses, dose and response relationships, dose-response models, and model constants. The first twenty items retrieved were all read and five appropriate ones were downloaded for thorough reading. The search was stopped when necessary information was found.

*Risk Characterization.* The last step was risk characterization which involved integrating all the steps taking into account uncertainties and severity of adverse effects [15]. Risk characterization was carried out to integrate hazard identification, exposure assessment, and the dose-response relationship to determine the risk of infection [47]. The risk for each simulated scenario was then calculated using the exponential model and Beta Poisson model for helminths and the bacterial pathogens (*E. coli* and* Salmonella*), respectively [24, 52]. The dose-response relationships were estimated using the two semimechanistic models of the infection process depending on pathogenic microorganism under consideration [53]. It has been reported that helminths to host interactions follow an exponential model where a single organism may lead to infection:

(1) $$\begin{array}{c} P_{inf}=1-e^{-rd}. \end{array}$$

*E. coli* and* Salmonella* host interaction have been found to follow a Beta Poisson model. The model assumes that the probability of infection increases with dose. The model is approximated by

(2) $$\begin{array}{c} P_{inf}=1-{\left\{\;1+\left(\;\frac{d}{\beta }\;\right)\;\right\}}^{-\alpha }, \end{array}$$

where *P*_inf_ is the probability of infection, *d* is the ingested dose, *α* is the pathogen constants, *β* is the dose-response parameters, and *r* is the probability of one organism initiating an infection [23]. The dose-response parameters used for* Salmonella* were *N*_50_ = 23600 and  *α* = 0.3126 and for* E. coli N*_50_⁡ = 1120 and  *α* = 0.2099 while they were *r* = 1 for helminths [54].

### 2.3. Ethical Consideration

An informed consent was obtained from household heads where a latrine was selected for inclusion in the study. During observations, consent was obtained from the same household heads and all households who did not consent to the study were not included and those who consented were free to discontinue with the study at any time. Names of households were anonymized and kept confidential. The study received ethical approval from the University of Malawi, College of Medicine Research Ethics Committee, in October 2014 (P.04/14/1565).

## 3. Results

### 3.1. Hazard Identification

#### 3.1.1. Concentration of Pathogens in Sludge from EcoSan Latrines

Identification of microbiological hazards involved measuring the presence of viable STHs and colony forming units per gram of sludge bacterial pathogens. In total, 35 latrines were sampled comprising 13 FAs and 22 UDDTs. The hazards targeted and identified included* Ascaris lumbricoides*, hookworms,* Taenia*,* E. coli,* and* Salmonella*.

The average concentration of viable* Ascaris lumbricoides* was 0.40 and 0.39 potentially viable eggs per gram of sludge from FAs (*n* = 13) and UDDTs (*n* = 22), respectively (Table 1). The *t*-test showed that there were more hookworms in sludge from FAs than in UDDTs (*p* = 0.001). The FAs were sampled in rural areas while UDDT was from the periurban areas in Malawi.

### 3.2. Exposure Assessment

**Figure 4:**
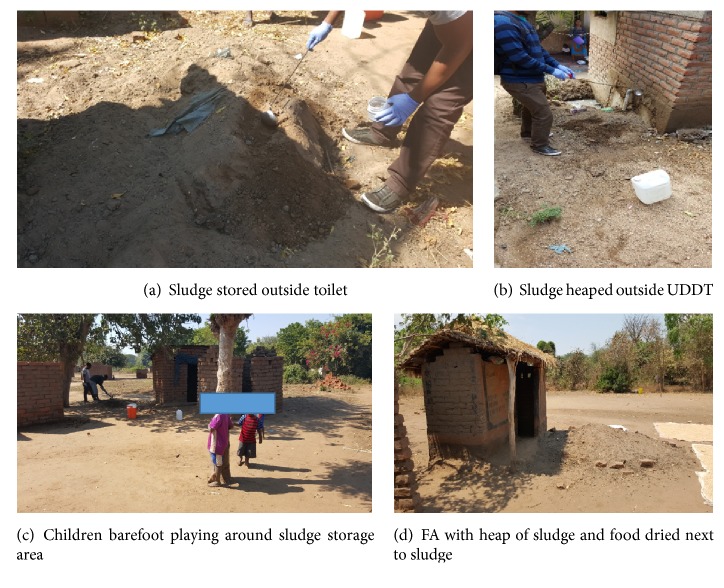
Faecal sludge storage and possible exposure pathways.

All 35 households with EcoSan latrines were observed for practices while 2 FAs and 1 UDDT were observed during emptying. The main sources of microbial hazards were identified through observations and were from inhalation of aerosols and dust, accidental ingestion and through contact with faecal sludge and through skin penetration in the case of hookworms. All members of the family who use EcoSan sludge were at risk of exposure to the sludge. During observations, sludge was seen spread around by the animals and chicken reared at the household. Sludge that spread around the household surroundings was likely to contaminate foodstuffs, for example, maize flour and other foods like rice and maize which were dried around the same location (Figure 4(d)). After harvesting, the sludge was heaped outside the latrine and eventually spreads around the household area. Two households using UDDTs in Blantyre kept their sludge in sacks. As also reported elsewhere, during emptying, personal protective equipment like gloves, plastic papers, and gumboots/shoes was rarely used and since gloves are not commonly available in rural areas of Malawi, EcoSan users were encouraged to be using plastic papers like empty sugar packets as gloves [18]. Figures 4(a), 4(c), and 4(d) show where EcoSan sludge is usually kept after harvesting.

In order to approximate the amount of EcoSan sludge ingested by people, men aged between 20 and 50 years owning and using EcoSan toilet were used to estimate exposures doses as it was difficult to approximate exposure doses for every member of the household. In rural areas of Malawi, men are often in charge of building toilets, emptying them, transporting EcoSan sludge to gardens, and also applying of the EcoSan sludge in the gardens. The following formula was used to approximate the sludge dose:

(3) $$\begin{array}{c} EDY=EEDD\times ETW\times EW\times EM, \end{array}$$

where EDY = estimated exposure dose of sludge in grams per person per year; EEDD = estimated exposure dose in grams per person per day; ETW = exposure time in days per week; EW = exposure weeks per month; EM = exposure months per year.

Schönning et al. (2007) reported that children ingest approximately 0.2 g of soil per day while adults were estimated to ingest about 15 to 50% of the children's dose (0.03 to 0.1 g) of soil per day [14]. We used 0.1 g per day as maximum ingested and inhaled dose per day. Exposure through dermal contact was estimated at three times that for ingestion and inhalation (0.3 g per day) due to the observed practices of not wearing gloves and shoes during emptying and application in the field of the sludge. The exposure time was higher for those using FAs in rural areas because all the users observed had no gloves and shoes to use during emptying. The estimated doses of sludge ingested and inhaled and through dermal contact per person per year for FAs and UDDTs are shown in Table 2.

Each man was estimated to be exposed to an average of 21.2 g and 12.8 g of EcoSan sludge from FAs and UDDTs, respectively, in a year (Table 2). The pathogen doses per person per year were estimated by multiplying the mean concentration of pathogens per gram by the estimated sludge dose ingested per year (Table 3).

It is estimated that each individual exposed to EcoSan sludge will have ingested and inhaled through dust about 1.3 and 0.8 potentially viable eggs of* Ascaris lumbricoides* per year if they are using sludge from FA and UDDT, respectively (Table 3).

### 3.3. Hazard Characterization

The adverse health effects for the organisms identified were obtained from literature and included diarrhoea and infection with helminths. Previous studies done in Malawi also indicated the existence and prevalence of these pathogens [57–59], hence this assessment. Hazard characterization used data from exposure assessment and literature to produce dose-response curves. The dose-response relationships gave an estimate of the adverse health effects in relation to the microbial dose ingested. The minimum infectivity doses are the number of pathogenic organisms ingested by a person which are able to cause infection. The infectivity doses for pathogens considered are shown in Table 4.

The infectivity doses outlined in Table 4 were estimated from data obtained on exposures to volunteers and from outbreak data. During these investigations, it was found that helminths to host interactions follow an exponential model where a single organism may lead to infection while* E. coli* and* Salmonella* host interaction were found to follow a Beta Poisson model.

This study used an online dose-response visualization and modelling application to come up with response levels for estimated pathogen doses [60]. Helminths have been shown to have a high response rate even with less doses (Table 5) because of the ability for a single viable ovum to cause infection in a human being while bacterial pathogens mainly depend on the number of pathogenic organisms ingested and their ability to survive in the host [61].

### 3.4. Risk Characterization

Risk characterization was carried out to integrate hazard identification, exposure assessment, and the dose-response relationship to determine the risk of infection. Exposure sludge doses were simulated to reduce uncertainty. The average simulated sludge doses were multiplied by the mean pathogen concentration to obtain an average pathogen dose ingested by a single person.  Table 6 presents the health risks as a result of exposure to EcoSan sludge from FAs and UDDTs from rural and periurban areas, respectively.

The estimated risk was calculated by using the exponential model for* Ascaris lumbricoides, Taenia, Trichuris trichiura, *and hookworms while for* E. coli *and* Salmonella, *a Beta Poisson model was used. All the calculations were done in Microsoft Excel. It was shown that hookworms presented the highest risk of approximately 1. The hookworm exposure was for dermal contact, inhalation, and ingestion. This meant that the risk for hookworms among EcoSan users was approximately 100% (Table 6).

## 4. Discussion

The viable hookworm eggs per gram of sludge were significantly higher in rural areas than in periurban areas (10.1 against 5.2 viable eggs per gram of sludge, *p* = 0.001). Sludge from the two locations were not different in terms of potentially viable* Ascaris eggs* (0.40 versus 0.39 viable eggs per gram of sludge, *p* = 0.84). This implies that the sludge quality has improved considering the helminth levels that were found in other past studies from developing countries which were in the range of 20 to 735 viable helminth ova per gram [23, 33, 38]. This could be due to a reduction of helminthic infections in the population attributable to the various interventions including mass drug administration which has been proved to reduce helminth levels in some populations [62, 63]. However, still more needs to be done to reduce the levels from the current 9.4 potentially viable eggs per gram to less than one as required by WHO and the US EPA standard of less than one ova per 4 g of sludge [34, 64].

The colony forming units for* Salmonella* were higher for UDDTs than for FAs (509.1 versus 346.2 CFUs, *p* = 0.35) while that for* E. coli* was similar in FAs and in UDDTs (1007.7 versus 859.1 CFUs, *p* = 0.79). The differences in* Salmonella* levels could be because of the differences in concentration of organisms and the type of EcoSan latrines in the two districts. This could also be due to weather patterns as FAs were sampled from Chikwawa (average of 40°C) where temperatures are most of the times higher than Blantyre (average of 26°C) where the UDDTs were sampled [65]. Suspected* E. coli* and* Salmonella* colony forming units were higher than the US EPA standard of less than 100 and none per 50 g, respectively, for class A biosolids [64]. However, the* E. coli* levels were within standard for the WHO guideline for unrestricted use of excreta in agriculture of less than 1000 organisms per gram of sludge [37]. Overall, this means the sludge requires further treatment if it is to be used without posing any health risk.

The observations showed that every individual is exposed differently depending on how they empty the pits, store the sludge, and use the sludge and whether they wear shoes. Those that store their sludge in sacks and use personal protection during emptying and applying in the field are exposed to less doses of sludge because sludge does not spread around the household surrounding and protection reduces contact with sludge, respectively. In addition, the quality of sludge is an important factor on the effects of exposure. This study considered an average man aged between 20 and 50 years who owns and uses an EcoSan toilet as a reference person for the risk assessment. The main exposure pathways were inhalation ingestion and dermal contact. During interaction with EcoSan users, it was observed that building of toilets, emptying, and transportation are mainly done by men while applying in the field was done by all adult members of the family. During in-depth interviews whose results were published elsewhere, it was found that some families delayed to empty their latrines because the men were busy [18]. It was therefore appropriate to estimate the health risk to men when in contact with EcoSan sludge.

Using Beta Poisson model, we estimated the risk for using EcoSan sludge. The risk for* Ascaris lumbricoides* alone was approximated at a minimum of 5.6 × 10^−1^ for using EcoSan sludge from both FAs and UDDTs. The risk of 5.6 × 10^−1^ means that about 6 out of 10 (60%) people using FAs or UDDTs will be infected by* A. lumbricoides* during a year or at an individual level; it means that out of ten exposures to sludge 6 times will result in infection. The risk obtained was above the WHO recommendation of between 10^−4^ and 10^−3^ infections per year [34]. The minimum risk for* Salmonella *and* E. coli *was 8.9 × 10^−2^ which means at least 9 people out of 100 (9%) will get infected. The risk for helminths and* Salmonella* found in this study was higher than the that found for accidental ingestion of faecal matter treated with 4% of urea in Uganda which was found to be 1 × 10^−3^ [66]. This may be because risk in this study was for both ingestion and inhalation. Furthermore, the higher risk could be due to specific practices of users during emptying, storage, transportation, and use in the field and assumptions made on duration of exposure. Users of EcoSan in the study areas rarely use protection and they store the faecal sludge after emptying in open spaces behind their households and allow animals to spread the sludge around the household thereby increasing the risk especially if the sludge contains pathogens [18]. As expected the risk was also higher than that found in Denmark which found a risk of 6 × 10^−11^ from handling human faeces from UDDT after 12 months of storage which may be attributed to less chances of infection from environmental risks due to weather conditions and low prevalence of helminths among the population [14]. Similarly, the risks for* E. coli* and* Salmonella* were also higher than the WHO acceptable limits [34]. Though children were not the main focus they have been found to be highly exposed to sludge due to their behaviour of playing outside where sludge is stored and also the tendency of walking barefoot. It is also reported elsewhere that doses of children are twice adult doses; hence their risks are expected to be more than what we found in our study [14]. This calls for action to reduce the risks through management of exposure routes especially helminths which have high chances of reinfecting their hosts. Further research should concentrate on reducing uncertainties in estimating the dose of soil ingested by each person and also on whether the organisms ingested will result into infection.

## 5. Conclusions

We present a health risk estimate for sludge use from FAs and double vault urine diverting dry latrines (UDDTs) found in Malawi. The assessment will assist public health officers in designing effective intervention aimed at reducing the risks that users of EcoSan latrines face in Malawi. Promoters of EcoSan latrines need to consider advocating for strict guidelines on EcoSan sludge use. Users should properly store their sludge; that is, in sacks, children should not be allowed to play where sludge is kept and consider using personal protection during emptying the pits and applying the sludge in the gardens in order to reduce the health risks.

## Figures and Tables

**Table 1:** Mean pathogen concentrations in sludge from FA and UDDT.

	Mean	Standard deviation	Range	Mean	Standard deviation	Range
Helminths	Concentration range, FAs (potentially viable eggs per gram of sludge), *n* = 13			Concentrations range, UDDTs (potentially viable eggs per gram of sludge), *n* = 22		

*A. lumbricoides*	0.40	0.74	0–2.42	0.39	0.58	0–1.87
*Hookworm*	10.10	13.5	0–17.23	5.2	4.61	0–17.27
*Trichuris trichiura*	0	0	0	0	0	0
*Taenia*	0.21	0.47	0–1.61	0.30	0.65	0–2.61

Bacterial pathogens	Colony forming units per gram (CFUs/g)			Colony forming units per gram (CFUs/g)		

*Salmonella*	346.2	405.4	0–1200	509.1	741.9	0–3200
*E. coli *	1007.7	1302.2	0–4100	859.1	1230.4	0–5500

**Table 2:** Estimated exposure doses of EcoSan sludge.

Exposure route	How people may get exposed	How much and for how long	Estimated exposure days in a year		Estimated dose range in g per year		Estimated average doses in g per year	
			FA	UDDT	FA	UDDT	FA	UDDT
Inhalation	Inhalation through aerosol in the air while walking outside where humus is stored, farming, emptying, and application in the field	0.1 g maximum per day for 6 months in a year, approximately 5 and 3 days a week in rural (FA) and periurban areas (UDDT), respectively	120	72	0–6	0–3.6	3.0	1.8

Ingestion	Accidental ingestion during pit emptying, transportation, application in the field without personal protection, and limited hand washing and through water and food contamination	Estimated at 0.1 g maximum per day for 4 days a year	4	4	0–0.4	0–0.4	0.2	0.2

Dermal contact	Skin contact through bare feet around household where humus is stored, emptying, and transportation and during application in the field	Estimated at 0.3 g maximum per day for 6 months in a year, approximately 5 and 3 days a week in rural (FA) and periurban areas (UDDT), respectively	120	72	0–36	0–21.6	18.0	10.8

**Table 3:** Estimated average dose of organisms ingested, inhaled, ingested, and exposed through dermal contact.

Pathogen	Exposure route	FAs			UDDTs		
		Mean eggs per gram	Sludge dose (g) per person per year	Pathogen dose per person per year	Mean eggs per gram	Sludge dose (g) per person per year	Pathogen dose per person per year
*Ascaris lumbricoides*	Ingestion and inhalation	0.40	3.2	1.3	0.39	2.0	0.8
*Taenia*	Ingestion and inhalation	0.21	3.2	0.7	0.30	2.0	0.6
Hookworms	Dermal contact, inhalation, and ingestion	10.10	21.2	214.1	5.2	12.8	66.6

Bacteria (CFUs per gram)							
*E. coli *	Ingestion and inhalation	1007.7	3.2	3224.6	859.1	2.0	1718.2
*Salmonella*	Ingestion and inhalation	346.2	3.2	1107.8	509.1	2.0	1018.2

**Table 4:** Minimum infective doses for selected microorganisms.

Microorganism	Minimum infective dose (number of organisms)	Source
Helminth	10^0^ to 10^1^	Andreoli et al., 2007 [55]
*Salmonella *spp.	10^4^ to 10^7^	Mara and Horan, 2003 [56]
*Salmonella typhi*	10	Mara and Horan, 2003 [56]
*E. coli *0157:H7	10 to 10^2^	Mara and Horan, 2003 [56]

**Table 5:** Response levels for the estimated pathogens doses.

Number	Pathogen	Pathogen dose (viable eggs or CFUs per person per year)		Response levels	
		FAs	UDDTs	FAs	UDDTs
(1)	*A. lumbricoides*	1.3	0.8	0.73	0.55
(2)	*Taenia*	0.7	0.6	0.50	0.45
(3)	Hookworms	214.1	66.6	1.00	1.00
(4)	*E. coli*	3224.6	1718.2	0.60	0.54
(5)	*Salmonella*	1107.8	1018.2	0.10	0.90

**Table 6:** Estimated annual risk due to exposure to faecal sludge from EcoSan latrines.

Pathogen	Route of exposure	FAs		UDDTs	
		Average pathogen dose ingested	Estimated risk	Average pathogen dose ingested	Estimated risk
*Ascaris lumbricoides*	Inhalation and ingestion	1.28	7.2 × 10^−1^	0.83	5.6 × 10^−1^
*Taenia*	Inhalation and ingestion	0.65	4.8 × 10^−1^	0.58	4.4 × 10^−1^
Hookworms	Dermal contact, inhalation, and ingestion	220.3	1.0 × 10^0^	66.4	1.0 × 10^0^

Bacteria (CFUs per gram)					
*E. coli *	Inhalation and ingestion	3258.7	6.0 × 10^−1^	1654.3	5.4 × 10^−1^
*Salmonella*	Inhalation and ingestion	1122.4	1.0 × 10^−1^	1001.2	8.9 × 10^−2^

## References

[1] Nakagiri A., Niwagaba C. B., Nyenje P. M., Kulabako R. N., Tumuhairwe J. B., Kansiime F. (2016). Are pit latrines in urban areas of Sub-Saharan Africa performing? A review of usage, filling, insects and odour nuisances. *BMC Public Health*.

[2] Höglund C. (2001). *Evaluation of microbial health risks associated with the reuse of source-separated human urine*.

[3] Whipple G. C. (1914). The sewage disposal problem in villages and small cities. *American Journal of Public Health*.

[4] Hansen P. (1942). Sewage Disposal Problems at Army Camps. *American Journal of Public Health*.

[5] Grimason A. M., Davison K., Tembo K. C., Jabu G. C., Jackson M. H. (2000). Problems associated with the use of pit latrines in Blantyre, Republic of Malawi. *Journal of The Royal Society for the Promotion of Health*.

[6] Smet J., Sudgen S. WELL - Resource Centre Network for Water, Sanitation and Environmental Health. http://www.lboro.ac.uk/well/resources/fact-sheets/fact-sheets-htm/Ecological%20sanitation.htm.

[7] Humphries D. L., Stephenson L. S., Pearce E. J., The P. H., Dan H. T., Khanh L. T. (1997). The use of human faeces for fertilizer is associated with increased intensity of hookworm infection in Vietnamese women. *Transactions of the Royal Society of Tropical Medicine and Hygiene*.

[8] Kumwenda S., Msefula C., Kadewa W., Ngwira B., Morse T. (2014). Is manure from ecological sanitation latrines safe for use to fertilize crops? A review of evidence from literature. *Sustainable water and sanitation services for all in afast changing world*.

[9] CCODE (2011). *Re-Use of ecosan products in Malawi, Experiences from Users in Peri-Urban Areas*.

[10] Morgan P. (2013). *Mekonnen A Tesfaye. Paving the Way to Scaling Up Ecosan in Malawi*.

[11] Jiménez B. (2009). *Pathogens of concern in dry toilets from developing countries and risk of using the sludge produced in agriculture*.

[12] WHO (2009). *Global Health Risks: Mortality and Burden of Disease Attributable to Selected Major Risks*.

[13] Jiménez B., Austin A., Cloete E., Phasha C., Beltrán N. (2007). Biological risks to food crops fertilized with Ecosan sludge. *Water Science and Technology*.

[14] Schönning C., Westrell T., Stenström T. A. (2007). Microbial risk assessment of local handling and use of human faeces. *Journal of Water and Health*.

[15] Boone I., Van der Stede Y., Aerts M., Mintiens K., Daube G. (2010). Quantitative microbial risk assessment: methods and quality assurance. *Vlaams Diergeneeskd Tijdschr*.

[16] Ministry of Irrigation and Water Development (2011). *Low cost Latrine Techologies*.

[17] Morgan M. (2010). *Ecological Sanitation in Malawi - Putting recycling into practice*.

[18] Kumwenda S., Msefula C., Kadewa W., Ngwira B., Morse T., Ensink J. H. J. (2016). Knowledge, attitudes and practices on use of fossa alternas and double vault urine diverting dry (DVUDD) latrines in Malawi. *Journal of Water Sanitation and Hygiene for Development*.

[19] Jimenez B., Austin A., Cloete E., Phasha C. (2006). Using Ecosan sludge for crop production. *Water Science and Technology*.

[20] EPA US, Environmental Protection Agency (1992). http://www.iaea.org/inis/collection/NCLCollectionStore/_Public/27/056/27056989.pdf#page=293.

[21] Blumenthal U. J., Mara D. D., Peasey A., Ruiz-Palacios G., Stott R. (2000). Guidelines for the microbiological quality of treated wastewater used in agriculture: recommendations for revising WHO guidelines. *Bulletin of the World Health Organization*.

[22] Hoglund C., Stenstrom T. A., Ashbolt N. (2002). Microbial risk assessment of source-separated urine used in agriculture. *Waste Management & Research*.

[23] Navarro I., Jiménez B., Cifuentes E., Lucario S. (2009). Application of Helminth ova infection dose curve to estimate the risks associated with biosolid application on soil. *Journal of Water and Health*.

[24] Eisenberg J. N. S., Soller J. A., Scott J., Eisenberg D. M., Colford J. M. (2004). A dynamic model to assess microbial health risks associated with beneficial uses of biosolids. *Risk Analysis*.

[25] Yajima A., Koottatep T. (2010). Assessment of E. coli and Salmonella spp. infection risks associated with different fecal sludge disposal practices in Thailand. *Journal of Water and Health*.

[26] Scott R. W. Resource Centre Network for Water, Sanitation and Environmental Health. http://www.lboro.ac.uk/well/resources/Publications/Briefing%20Notes/BN27%20Ecological%20sanitation.htm.

[27] Odikamnoro O., Ike I. (2009). *Possible Public Health Implications of excreta re-use in poorly sanitated rural farming communities of Ebonyi State, Southeast Nigeria*.

[28] Itchon S. G., Holmer J. R., Tan M. L. B. (2009). *The Public Health Safety of Using Human Excreta from Urine Diverting Toilets for Agriculture: The Philippine Experience*.

[29] Nordin A. (2010). *Ammonia sanitisation of human excreta treatment technology for production of fertiliser*.

[30] Nordin A., Nyberg K., Vinnerås B. (2009). Inactivation of ascaris eggs in source-separated urine and feces by ammonia at ambient temperatures. *Applied and Environmental Microbiology*.

[31] Mehl J. A. (2008). *Pathogen destruction and aerobic decomposition in composting latrines: A study from rural panama*.

[32] Corrales L. F., Izurieta R., Moe C. L. (2006). Association between intestinal parasitic infections and type of sanitation system in rural El Salvador. *Tropical Medicine & International Health*.

[33] Nalivata P., Kadewa W. (2012). *Risks and benefits of using ecosan toilets. In*.

[34] (2006). *excreta and greywater*.

[35] Chipeta M. G., Ngwira B., Kazembe L. N. (2013). Analysis of schistosomiasis haematobium infection prevalence and intensity in Chikhwawa, Malawi: an application of a two part model. *PLOS Neglected Tropical Diseases*.

[36] Phiri K., Whitty C. J. M., Graham S. M., Ssembatya-Lule G. (2000). Urban/rural differences in prevalence and risk factors for intestinal helminth infection in southern Malawi. *Annals of Tropical Medicine and Parasitology*.

[37] Petterson SA., Ashbolt NJ. (2005). WHO guidelines for the safe use of wastewater and excreta in agriculture: Microbial risk assessment section. http://citeseerx.ist.psu.edu/viewdoc/download?doi=10.1.1.471.8530&amp;rep=rep1&amp;type=pdf.

[38] Jimenez B., Austin A., Cloete E., Phasha C. (2006). Using Ecosan sludge for crop production. *Water Science and Technology*.

[39] Jensen P. K., Phuc P. D., Konradsen F., Klank L. T., Dalsgaard A. (2009). Survival of Ascaris eggs and hygienic quality of human excreta in Vietnamese composting latrines. *Environmental Health: A Global Access Science Source*.

[40] Cassin M. H., Lammerding A. M., Todd E. C. D., Ross W., McColl R. S. (1998). Quantitative risk assessment for *Escherichia coli* O157:H7 in ground beef hamburgers. *International Journal of Food Microbiology*.

[41] Sunger N., Haas C. N. (2015). Quantitative microbial risk assessment for recreational exposure to water bodies in Philadelphia. *Water Environment Research*.

[42] Moodley P., Archer C., Hawksworth D., Leibach L. (2008). *South Africa, Water Research Commission. Standard methods for the recovery and enumeration of helminth ova in wastewater, sludge, compost and urine-diversion waste in South Africa*.

[43] ISO 6579 (2002). General guidance on methods for the detection of Salmonella. http://www.iso.org/iso/iso_catalogue/catalogue_ics/catalogue_detail_ics.htm?csnumber=12985.

[44] Rashid N., Misbahuddin M., Choudhry Z. K., Saleh A. A., Sattar N. I. (2014). The colony count of Escherichia coli in the stool of palmar arsenical keratosis following probiotics supplementation. *Bangladesh Journal of Pharmacology*.

[45] Cheesbrough M. (2006). *District Laboratory Practice in Tropical Countries*.

[46] McKinley J. W., Parzen R. E., Guzmán Á. M. (2012). Ammonia inactivation of Ascaris ova in ecological compost by using urine and ash. *Applied and Environmental Microbiology*.

[47] Katukiza A. Y., Ronteltap M., van der Steen P., Foppen J. W. A., Lens P. N. L. (2014). Quantification of microbial risks to human health caused by waterborne viruses and bacteria in an urban slum. *Journal of Applied Microbiology*.

[48] National Statistical Office Malawi Demographic and Health Survey 2015-16. http://www.dhsprogram.com/pubs/pdf/PR73/PR73.pdf.

[49] Gerba C. P., Smith J. E. (2005). Sources of pathogenic microorganisms and their fate during land application of wastes. *Journal of Environmental Quality*.

[50] United States Environmental Protection Agency (2017). Use of Monte Carlo Simulation in Risk Assessments. *United States Environmental Protection Agency*.

[51] Burmaster DE., von Stackelberg K. (1991). Using Monte Carlo simulations in public health risk assessments: estimating and presenting full distributions of risk. *Journal of Exposure Analysis and Environmental Epidemiology*.

[52] Hamilton K. A., Haas C. N. (2016). Critical review of mathematical approaches for quantitative microbial risk assessment (QMRA) of Legionella in engineered water systems: research gaps and a new framework. *Environmental Science: Water Research & Technology*.

[53] Fewtrell L., Bartram J., World Health Organization (2001). *Water quality: guidelines, standards, and health: Assessment of risk and risk management for water-related infectious disease*.

[54] Westrell T. (2004). *Microbial risk assessment and its implications for risk management in urban water systems*.

[55] Andreoli C. V., von Sperling M., Fernandes F. (2007). Sludge Treatment and Disposal. *Water Intelligence Online*.

[56] Mara D., Horan N. J. (2003). *Handbook of Water and Wastewater Microbiology*.

[57] Taulo S., Wetlesen A., Abrahamsen R., Kululanga G., Mkakosya R., Grimason A. (2008). Microbiological hazard identification and exposure assessment of food prepared and served in rural households of Lungwena, Malawi. *International Journal of Food Microbiology*.

[58] Phiri K. S. (2001). The prevalence, intensity and ecological determinants of helminth infection among children in an urban and rural community in southern Malawi. *Malawi Medical Journal*.

[59] Bowie C., Purcell B., Shaba B., Makaula P., Perez M. (2004). A national survey of the prevalence of schistosomiasis and soil transmitted helminths in Mala*ŵ*i. *BMC Infectious Diseases*.

[60] Center for Advancing Microbial Risk Assessment (2015). *Dose-Response Visualization and Modelling Application*.

[61] Leggett H. C., Cornwallis C. K., West S. A. (2012). Mechanisms of pathogenesis, infective dose and virulence in human parasites. *PLoS Pathogens*.

[62] Hodges M. H., Dada N., Warmsley A. (2012). Mass drug administration significantly reduces infection of Schistosoma mansoni and hookworm in school children in the national control program in Sierra Leone. *BMC Infectious Diseases*.

[63] Parker M., Allen T. (2011). Does mass drug administration for the integrated treatment of neglected tropical diseases really work? Assessing evidence for the control of schistosomiasis and soil-transmitted helminths in Uganda. *Health Research Policy and Systems*.

[64] (1997). *Environment Protection Authority. Use and disposal of biosolids products*.

[65] Department of Climate Change and Meteorological Services (2016). *Malawi Meteorological data. Blantyre, Malawi: Ministry of natrural resources, energy and environment*.

[66] Regli S., Rose J. B., Haas C. N., Gerba C. P. (1991). Modeling the risk from Giardia and viruses in drinking water. *Journal - American Water Works Association*.

